# A rare case of Candida parapsilosis endocarditis in a young healthy woman – case report

**DOI:** 10.1186/1749-8090-8-29

**Published:** 2013-02-22

**Authors:** Mijomir Pelemiš, Goran Stevanović, Lidija Lavadinović, Snežana Matić, Ivana Milošević, Miloš Korać, Svetislav Pelemiš, Milan Nedeljković, Milica Prostran

**Affiliations:** 1 Clinic for Infectious and Tropical Diseases, Clinical Center of Serbia, Faculty of Medicine, University of Belgrade, Bulevar oslobodjenja 16, 11000, Belgrade, Serbia; 2 Clinic for Cardiology, Clinical Center of Serbia, Dr Koste Todorovica 8, 11000, Belgrade, Serbia; 3 Clinic for Otorhinolaringology, Clinical Center of Serbia, Pasterova 2, 11000, Belgrade, Serbia; 4 Department of Pharmacology, Clinical Pharmacology and Toxicology, Faculty of Medicine, University of Belgrade, 11000, Belgrade, Serbia

**Keywords:** Native valve, Antifungal therapy, Post surgical treatment, Candidaemia, Candida endocarditis

## Abstract

**Abstract:**

Disseminated fungal infections are still rare conditions, mostly caused by *Candida spp*. during immunosuppression. Infection of immunocompetent individuals is uncommon. Endocarditis is a rare manifestation during candidaemia, mostly in patients with prosthetic valves. Affection of previously unaltered valves is uncommon.

**Case presentation:**

We presented a case of a young, previously healthy female patient with endocarditis, caused by *Candida parapsilosis*. The initial symptom, fever, was present four months before hospital admittance. She was febrile without other symptoms and during observation in a local hospital. After her condition deteriorated, she was transferred to the Institute for infectious and tropical diseases, Belgrade. Clinical findings on admission include petechial skin rash and moderate hepatosplenomegaly. Newly developed systolic murmur was noted, and *Candida parapsilosis* was isolated in multiple blood cultures. Echocardiography revealed 15 × 14 mm vegetations on the right aortic vellum.

She was treated with antifungal drugs (fluconasole, liposomal amphotericin B), and the affected valve was successfully replaced. The same strain of *Candida parapsilosis* was isolated from the intraoperative material of the valve.

There were no markers of immunosuppression or other conditions which could affect the immune system.

**Conclusion:**

After a prolonged period of treatment she was successfully cured, and she received a long-term intermittent suppressive fluconasole therapy for the time being.

## Background

Although disseminated fungal infections are more common today than before, they still remain rare conditions, mostly caused by *Candida spp*. *Candida parapsilosis* and *Candida tropicalis* are the most common causes in Europe [1,2]. Risk factors include immunosuppression (HIV, neutropenia, transplants, solid tumors), but infection of immunocompetent individuals is uncommon [1,3]. Candidaemia has a significant mortality rate, up to 44% [1,2].

Endocarditis is present in 5-25% of patients with candidaemia, mostly in patients with prosthetic valves [4,5]. Affection of previously unaltered heart valves is uncommon. During the past 20 years, there have been only three cases reported in Sweden [4]. Other authors also refer to *Candida* endocarditis as an extremely rare occurrence in patients with normal native cardiac valves [6,7].

## Case presentation

We will present a case report of a previously healthy twenty-three year old Caucasian female patient, from a higher social-culture level, living in Belgrade suburbs. She had a negative history data to preexisting diseases, drug use or any underlining conditions.

First symptom-fever, presented four months before hospital admittance, was treated with third generation cephalosporin (ceftriaxone 2,0 gr daily i.v.) and resolved within 5 days. During the next month she was febrile without other symptoms. During that time the patient was observed in a local hospital, until her condition deteriorated - with anemia, leucopenia and petechial skin rash, and she was transferred to the Institute for Infectious and Tropical Diseases, Belgrade, Clinical Centre of Serbia. Clinical findings on admission included discreet petechial skin rash, moderate hepatosplenomegaly, whereas other systems were unaffected, including normal heart sounds. Laboratory tests showed moderate elevation of erythrocyte sedimentation rate, intermediary anemia and leucopenia, with elevated C-reactive protein. (Table 1) *Candida parapsilosis*- sensitive to all systemic antifungal drugs was isolated in multiple blood cultures. The initial treatment included IV fluconazole 200 mg/12 h. During the third day of therapy, a newly developed systolic murmur was noted, presenting an indication for echocardiography. The patient was afebrile starting from that moment.

**Table 1:** Laboratory results during course of illness, and follow-up

Analysis*	**Admission day**	**Day 15**	**Day 30 (preoperatively)**	**Day 40 (8 days after operation)**	**Discharge day**	**After 3 year follow-up**
SE (mm/hour)	54	50	48	36	18	2
HgB (gr/l)	72	105	126	117	146	140
Er x10^12^/l	2,71	3,21	3,66	3,82	4,12	4,08
Le x10^9^/l	3,7	3,1	4,0	6,5	6,2	5,6
Tr x10^9^/l	218	317	298	321	251	253
fibrinogen (gr/l)	2,2	4,1	4,4	4,8	2,4	2,6
CRP (mg/l)	38	22	28	20	4	2
PT (sec)	14,5	13,2	12,9	16,8	17,8	18,9

Legend:

*SE- erythrocytes sedimentation rate during first hour, HgB – hemoglobin, Er – ery throcytes, Le – leucocytes, Tr – thrombocytes, CRP – C-reactive protein, PT – prothrombine time.

**Figure 1:**
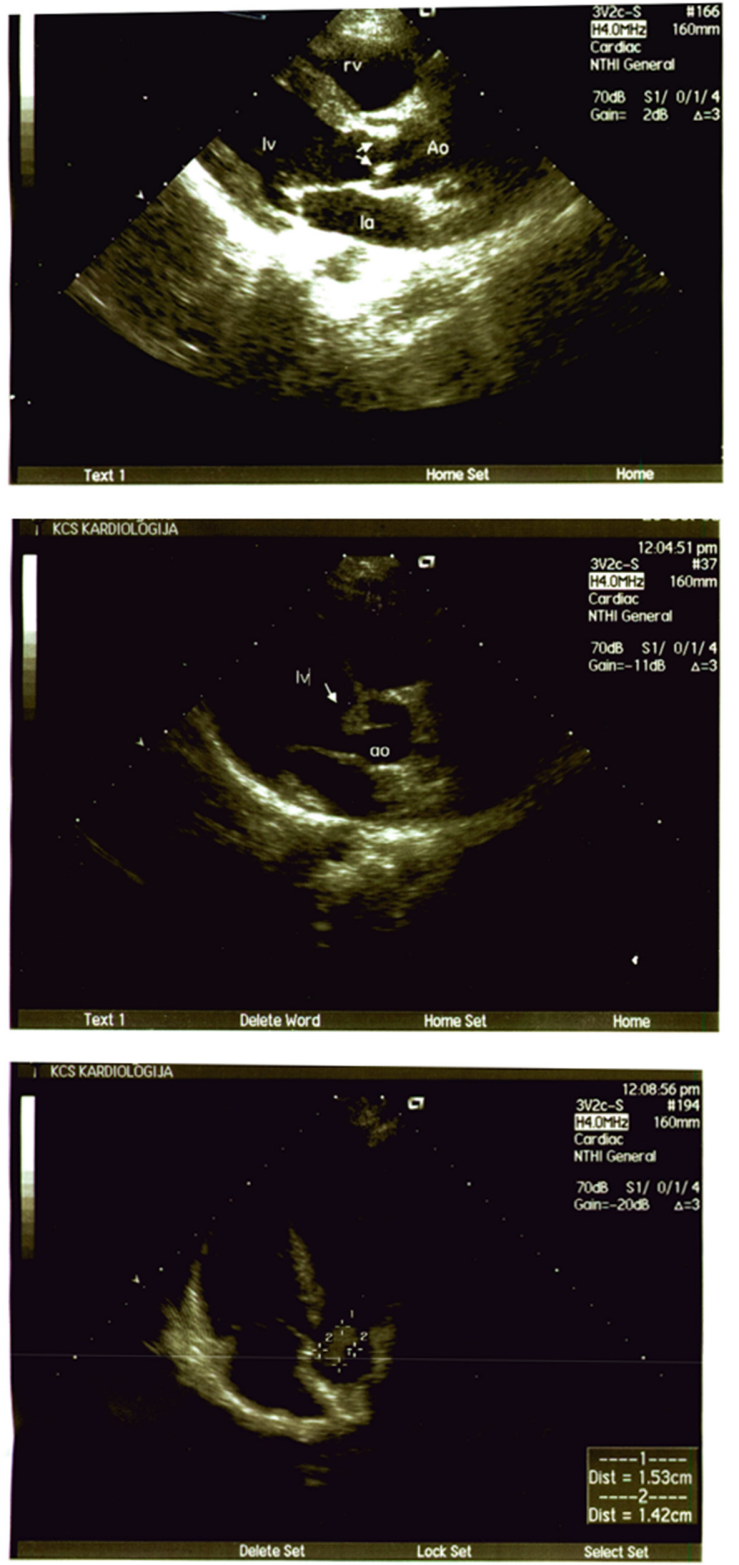
Transthoracal echocardiografy in longitudinal and apical 4 chamber view vegetations on the aortic valves.

**Figure 2:**
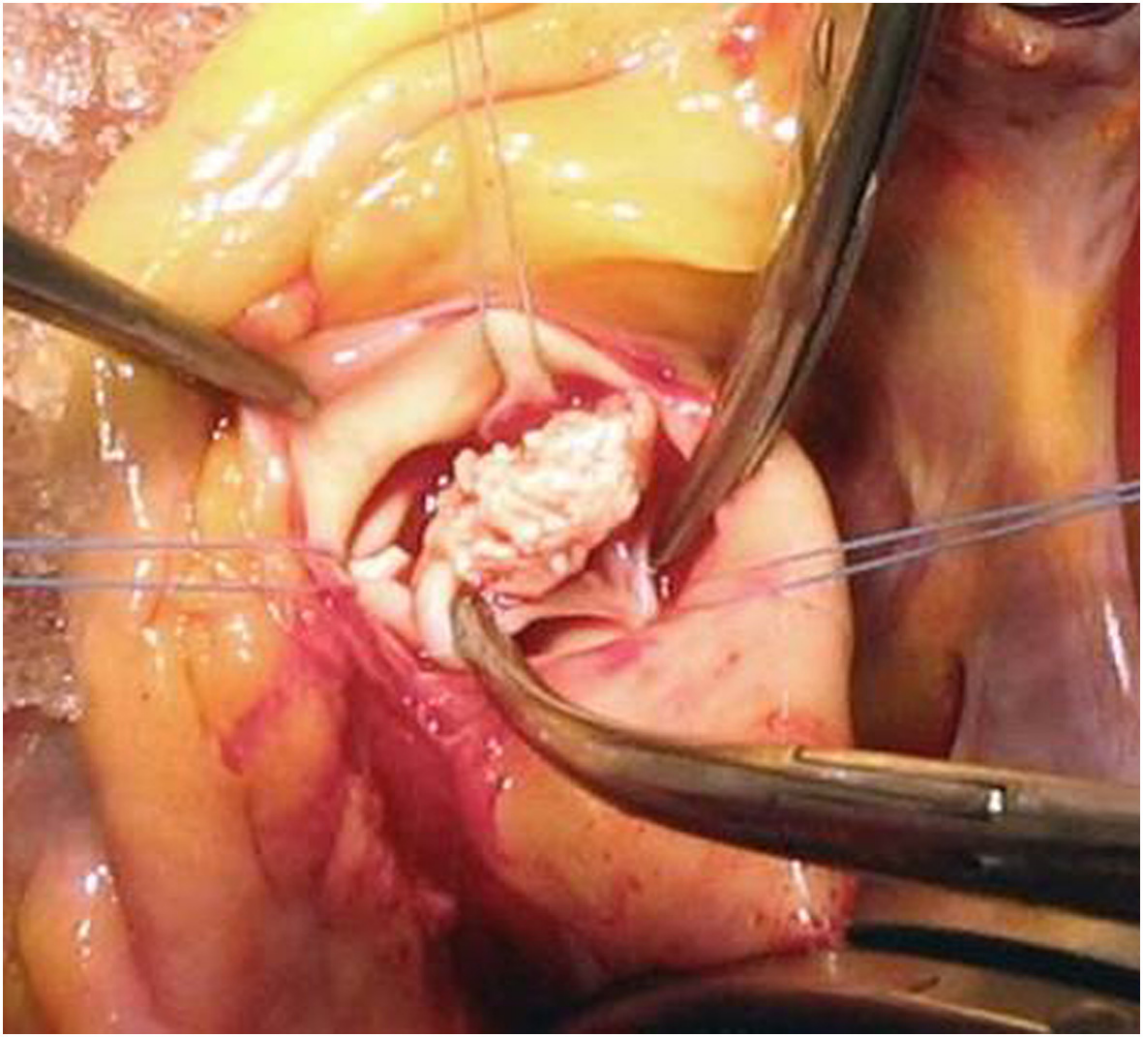
Intraoperaive vegetation findings - aortic valve.

Echocardiography revealed 15 × 14 mm vegetations on the right aortic vellum. (Figure 1) As Candida endocarditis usually appears in immunosuppressive patients as well as in patients with prosthetic valves, extensive clinical investigation was performed, to uncover any possible cause of immunosuppression. During a four-week period, all possible infectious agents were tested, including HIV, HBV, HCV, EBV, *Leischmania* and tuberculosis. Normal absolute count and ratio of CD4, CD2, CD3 and CD8 lymphocytes was noted, as well as normal concentration of immunoglobulines and sufficient immunological functions. There were no markers of autoimmune diseases which could affect the immune system. Hematological investigation was performed, as well as abdominal and chest CT, so the possible presence of solid tumors was excluded. A "10-panel urine screen" drug test was negative. During investigation, the patient was treated with fluconazole (200 mg/12 h, i.v.). Control echocardiography showed progressive enlargement of vegetations, spreading to the other vellum, so the treatment was continued with liposomal amphotericin B intravenously, 50 mg daily. Since the transoesophageal echocardiography showed that after two weeks of new treatment there were two additional vegetations affecting vellums (17 × 6 mm and 12 × 3 mm), it was necessary to replace the affected heart valve. Control blood cultures, repeated daily from the fifth day of hospitalization (patient was afebrile), were sterile. Preoperatively, patient was treated with fluconazole for 38 days, and liposomal amphotericin B for 15 days. The affected valve (Figure 2) was successfully replaced, and the same strain of *Candida parapsilosis* was isolated from intraoperative material of the valve.

Further conservative antifungal therapy was administered after operation, until laboratory findings were normalized, five months later. During this period the patient was treated with liposomal amphotericin B for 36 days, IV in the daily dose of 50 mg. For the rest of the period she was treated with fluconazole IV 200 mg in the dose intervals of 12 hours (approximately four months). During the whole period, no side-effects were observed.

During the 3 year follow-up, there were no other pathological developments, and the patient has been physically active and working.

## Discussion

This report presents a rare disease progress in a young and previously healthy person, without any predisposing conditions - no immunodeficiency or previous valve defect [4-6]. In spite of intensive systemic antifungal therapy, which is recommended [2,4,5] there was no improvement, so the operative valve replacement was necessary, which has also been reported in other cases [4-7].

After the patient was discharged from the hospital, she received a long-term intermittent suppressive fluconasole therapy. There is some difference in opinion concerning the time length of this therapy, including possibility of life-long treatment [5,8,9]. During the three year follow-up, our patient has been taking 200 mg of fluconasole twice a week with complete recovery and no other physical complaints.

## Conclusions

Although rare, Candida endocarditis in previously healthy patients, with no clear risk factors, is possible. During blood cultivation, one should always look for fungus. Candida endocarditis therapy is a combination of antifungal drugs and surgery, and the use of drugs is long-lasting.

## Consent

Written informed consent was obtained from the patient for publication of this case report and any accompanying images. A copy of the written consent is available for review by the Editor-in-Chief of this journal.

## Competing interests

The authors declare that they have no competing interests.

## Authors’ contributions

PM was the primary treating physician and is responsible for the treatment; SG participated in the treatment of patients and manuscript preparation; LL participated in the treatment of patients and manuscript preparation; MS was the main treating cardiologist who was constantly monitoring the patient; MI assisted in the treatment and monitoring of the patient and helped to draft the manuscript; KM assisted in the treatment and monitoring of the patient and helped to draft the manuscript; PS assisted in the treatment and monitoring of the patient and helped to draft manuscript; NM was the chief cardiologist responsible for echocardiogaphy and preoperative preparation; PM was the clinical pharmacologist responsible for the selection of antifungal drugs and their combination, as well as for the monitoring of adverse effects. All authors read and approved the final form manuscript.
